# Viral Metagenomic Sequencing Reveals Viral Codetection Patterns in Children with Respiratory Syncytial Virus Infection

**DOI:** 10.3390/v18091041

**Published:** 2026-09-19

**Authors:** Wenzhuo Ran, Xiaozhou He, Deng Luo

**Affiliations:** 1 Department of Laboratory Medicine, Traditional Chinese and Western Medicine Hospital of Wuhan, Tongji Medical College, Huazhong University of Science and Technology, Wuhan 430023, China; rwz5216@whyyy.com; 2 National Institute for Viral Disease Control and Prevention, Chinese Center for Disease Control and Prevention, Beijing 102206, China; 3 Department of Endocrinology, Renmin Hospital, Wuhan University, Wuhan 430060, China

**Keywords:** viral metagenomic sequencing, viral codetection, virome, respiratory syncytial virus, respiratory tract infection

## Abstract

Respiratory syncytial virus (RSV) is a leading cause of pediatric respiratory infections, yet the frequency and composition of viral codetections in affected children have not been extensively profiled using unbiased sequencing approaches. Here, we performed viral metagenomic sequencing on nasopharyngeal swabs collected from 42 pediatric patients with confirmed RSV infection to characterize the codetection landscape. RSV was detected and genotyped in all specimens. Beyond RSV, we identified nine additional human respiratory viruses coexisting across the cohort; several of these fall outside the scope of routine diagnostic panels, although their clinical significance remains uncertain. Codetections were the rule rather than the exception: 30 of 42 samples harbored at least three viruses, with up to seven distinct viruses detected in a single case. These findings underscore the taxonomic breadth and polymicrobial complexity of respiratory infections in children. Our results describe the viral codetection landscape in RSV-infected children, revealing a broader spectrum of co-occurring viruses than typically captured by targeted diagnostics, although the clinical significance of most codetections remains uncertain. These findings highlight the polymicrobial complexity of RSV infections and underscore the need for prospective studies to determine which codetections are clinically meaningful.

## 1. Introduction

Clinical diagnosis still assumes that one pathogen causes one disease. This assumption is convenient, but it hides how often several pathogens are present at once, and it affects how we interpret disease and decide on treatment [1,2,3]. Co-detection is not rare, and pathogens interact in ways that can change virulence, alter host defenses, and make the underlying cause hard to identify; viral interference and competitive suppression are two well-known examples [4,5]. Virus–virus interactions during respiratory coinfection can modulate disease severity through mechanisms such as viral interference and immune modulation [6]. When closely related viruses infect the same host, genetic exchange becomes possible and recombinant strains can emerge [7]. The central difficulty is that true coinfection and bystander co-detection are indistinguishable in a single specimen.

Mixed infections are rarely considered in the clinic unless the presentation is unusual, such as high mortality, neurological symptoms, or clear immunosuppression, and one pathogen cannot explain the findings [8]. Conventional tools are of limited help. Serology is insensitive and cross-reacts among related viruses, while culture depends on permissive cell lines or animal models, and when several viruses are present, isolating any one of them becomes harder. Culture-based studies before the molecular era could recover more than one virus from a specimen using multiple cell lines and varied conditions, though at much lower rates than molecular methods do today [9]. This difference is not simply a matter of sensitivity. Culture detects replicating virus; molecular methods detect nucleic acid, including non-viable virions, leftover RNA or DNA from past infections, and possibly commensal or transient colonizers. The lower co-detection rates in early culture studies may therefore mean that fewer viruses are actually active than nucleic acid data suggest.

PCR, qPCR, and targeted sequencing have made mixed infections much easier to detect, but they remain limited by their reliance on known sequences, which is precisely the gap that unbiased sequencing is meant to fill [10]. Multiplex PCR panels have shown that respiratory viral co-detections are common in children, yet because these panels detect only known targets, unexpected or poorly characterized viruses are missed. A recent narrative review of pediatric acute respiratory infections reported that the co-detection rate of multiple viruses ranges from 0.07% to 55%, and that multi-virus coinfection does not appear to increase disease severity, likely due to viral interference and immune modulation [11]. Metagenomic approaches have started to close this gap elsewhere, but the viruses that accompany RSV, the leading cause of pediatric respiratory hospitalization, have not been systematically surveyed without a priori target selection.

One caveat applies throughout: finding a viral nucleic acid sequence in a respiratory specimen does not prove active infection, because viral RNA or DNA can persist for weeks after an acute episode, and some viruses, such as *Human bocavirus* and *Anelloviruses*, are frequently detected in asymptomatic children [12,13]. A positive result may therefore reflect true coinfection, a sequential infection, or incidental carriage, and this distinction is clinically relevant. Lim et al. [14] found that viral co-detection was not associated with higher risks of fever, ICU admission, oxygen use, mechanical ventilation, or abnormal chest radiographs compared with single-virus detection, which suggests that illness is often driven by one dominant virus. We therefore use “co-detection” for the presence of multiple viruses in a specimen, and reserve “co-infection” for cases in which active concurrent infection can be established with more confidence [8].

Viral metagenomic sequencing has become a useful tool in diagnostics and public health [15]. It does not presuppose what to look for, and it captures more viruses than targeted assays, which has changed how we view the human virome [16]. It also helps trace viral evolution, discover new viruses, and characterize virus–host interactions, information relevant to vaccine and antiviral development [17], and it has been used clinically, including in pediatric central nervous system infections [18]. Its limitations are substantial. Sensitivity depends on host background, contamination, and sequencing depth, and amplification bias can distort abundance. More importantly, metagenomic data show that viral sequences are present but cannot tell whether the viruses are replicating, transcriptionally active, or infectious [19]. Because of these limitations, the viruses detected in this study are described as co-detections rather than as confirmed coinfections.

Respiratory tract infections are among the most common pediatric illnesses, and RSV accounts for a large share of hospitalizations in young children. Here, we used viral metagenomic sequencing to profile the viruses that co-occur with RSV in children and to examine what this approach adds beyond routine diagnostic panels.

## 2. Materials and Methods

### 2.1. Specimen Collection and RSV qPCR Confirmation

The institutional review board of Renmin Hospital, Wuhan University approved this retrospective study (approval no. [2026ACCR-M245]) and waived the informed consent requirement. We retrieved 42 nasopharyngeal swab specimens from pediatric patients whose initial clinical workup—by multiplex PCR panel or rapid antigen test—had flagged RSV infection at Renmin Hospital, Wuhan University between November 2025 and May 2026. The original laboratory reports, de-identified demographic records (age and sex), and clinical diagnoses were documented for downstream analyses.

Viral nucleic acids were extracted from 200-μL aliquots of each specimen with the QIAamp MinElute Virus Spin Kit (Qiagen, Hilden, Germany; cat. no. 52906) and eluted in 60 μL of AVE buffer. To confirm RSV status, we performed quantitative real-time PCR using the TaqMan^TM^ Fast Virus 1-Step Master Mix (Applied Biosystems, Foster City, CA, USA; cat. no. AM1005) on a QuantStudio 5 platform. Each 20-μL reaction contained 400 nM of forward and reverse primers and 200 nM of the TaqMan probe; the primer and probe sequences were taken from a previously published report [20]. Amplification proceeded at 95 °C for 15 s, followed by annealing/extension at 60 °C for 30 s, over 45 cycles. The RNase P gene served as an internal control to monitor sample quality and extraction efficiency. A cycle threshold (Ct) ≤ 38 was considered RSV-positive, whereas Ct > 38 or undetermined readings were scored as negative; this cut-off was used to stratify samples in subsequent codetection analyses.

### 2.2. Overview of the Study Workflow

**Figure 1:**
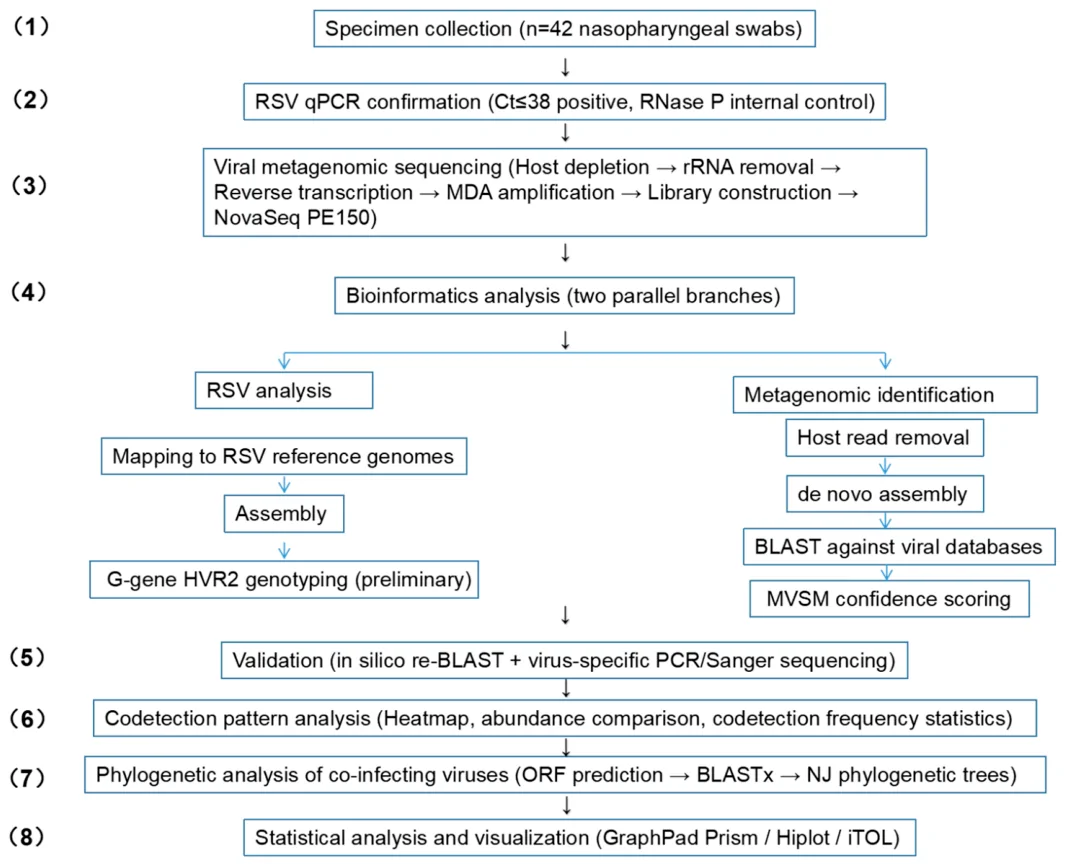
Schematic diagram of the study workflow. The workflow comprised eight main stages: (1) specimen collection from 42 RSV-positive pediatric patients; (2) qPCR confirmation of RSV; (3) viral metagenomic sequencing; (4) bioinformatics analysis, including parallel RSV genome assembly/genotyping and viral metagenomic identification; (5) in silico and experimental validation; (6) codetection pattern analysis; (7) phylogenetic analysis of co-infecting viruses; and (8) statistical analysis and visualization. Arrows indicate the sequential order of the procedures. RSV genotype assignments were performed with reference to the standardized classification framework proposed by Goya et al. [21]; however, as the present analysis was based on the G gene HVR2 region, definitive lineage confirmation would require full G gene or whole-genome sequencing.

To provide a comprehensive overview of the experimental and analytical procedures, the overall workflow of this study is illustrated in Figure 1.

### 2.3. Viral Metagenomic High-Throughput Sequencing

To deplete host-derived nucleic acids, the extracted total nucleic acids were first treated with DNase I (New England Biolabs, Ipswich, MA, USA; cat. no. M0570S) at 37 °C for 30 min to remove residual genomic DNA, followed by heat inactivation at 65 °C for 10 min. Ribosomal RNA (rRNA) depletion was then performed using the QIAseq FastSelect RNA Removal Kit (Qiagen, Hilden, Germany; cat. no. THIS-001Z) according to the manufacturer’s protocol. The RNA was reverse-transcribed into cDNA using the SuperScript^TM^ IV First-Strand Synthesis System (Invitrogen, Thermo Fisher Scientific, Waltham, MA, USA; cat. no. 18091050) with random hexamer primers at 50 °C for 10 min, followed by 80 °C for 10 min for enzyme inactivation. Non-targeted amplification of cDNA was achieved by multiple displacement amplification (MDA) using the REPLI-g Cell WGA & WTA Kit (Qiagen; cat. no. 150345) at 30 °C for 2 h, with heat inactivation at 65 °C for 5 min. We acknowledge that random-primed MDA can introduce amplification bias and may distort the relative representation of viral genomes, particularly for low-abundance or GC-rich templates. Therefore, viral abundance data in this study should be interpreted as semi-quantitative rather than absolute. To monitor potential reagent and environmental contamination, a negative control (nuclease-free water) was processed in parallel with the clinical specimens through nucleic acid extraction, DNase treatment, reverse transcription, and MDA amplification. As this control contained no amplifiable nucleic acid template, no library was obtained after library preparation, and it was therefore not subjected to sequencing. In addition, selected viral detections were experimentally confirmed by virus-specific PCR and Sanger sequencing, providing independent validation of the MDA-based workflow. Amplified products were purified with 0.6× volumes of DNA purification magnetic beads (VAHTS, Nanjing Vazyme Biotech Co., Ltd., Nanjing, China; cat. no. N411), washed twice with 75% ethanol (*v*/*v*), and eluted in 50 μL of nuclease-free water. Purified DNA was quantified using a Qubit 4.0 Fluorometer with the Qubit dsDNA HS Assay Kit (Thermo Fisher Scientific; cat. no. Q32851). DNA libraries were constructed by Novogene Co., Ltd. (Beijing, China) using the NEBNext Ultra II DNA Library Prep Kit (New England Biolabs) and subjected to paired-end (150 bp × 2) high-throughput sequencing on the NovaSeq 6000 platform (Illumina, San Diego, CA, USA), targeting approximately 3 Gb of raw data per sample. Only DNA samples and libraries that passed the standard quality control (QC) criteria of Novogene Co., Ltd. were used for library construction and sequencing. In brief, DNA samples were quality-controlled before library preparation, including quantification by Qubit, integrity assessment by agarose gel electrophoresis and capillary electrophoresis, and purity assessment for RNA, protein, and other contamination.

### 2.4. RSV Genome Assembly and Phylogenetic Analysis

Raw reads were quality-filtered using fastp (v0.23.4) to remove adapter sequences and low-quality bases (Q < 20), yielding clean reads. This threshold was chosen in accordance with common practice in viral metagenomic studies, where sufficient read coverage is required for reliable pathogen detection [22,23]. Clean reads were aligned to the RSV-A reference genome (GenBank: MT506703.1) and the RSV-B reference genome (GenBank: OP975389) using Bowtie2 (v2.3.5.1) under the defaultsensitive mode. The alignment files were converted, sorted, and indexed using SAMtools (v1.17), then imported into Geneious Prime (v2024.0.2) for consensus genome generation. For each sample, the number of RSV-mapped reads, mean sequencing depth, and the percentage of the reference genome covered (% Ref_seq) were calculated. Samples with a mean depth ≥ 10× and coverage ≥ 70% were retained for downstream genotyping.

For genotype assignment, the second hypervariable region (HVR2) of the G gene—defined as ≥90% coverage of the HVR2 reference region without ambiguous bases (Ns)—was extracted from the assembled genomes for phylogenetic analysis. We acknowledge that the standardized phylogenetic classification framework recently published by Goya et al. [21] represents the current gold standard for RSV genotype assignment below the subgroup level, which recommends the use of at least the full G gene ectodomain or complete G gene sequences for robust phylogenetic classification. However, as our sequencing strategy was primarily designed for metagenomic profiling of viral codetection rather than full-length RSV genome recovery, the HVR2 region was the most consistently recoverable region across the majority of samples. Therefore, our genotype designations should be interpreted as preliminary. These sequences were aligned using MAFFT (v7.526) against a reference panel representing all major RSV genotypes of both subtypes, including OR666591 (subtype B) and MT506703.1 (subtype A, reference genome), along with additional reference sequences from previously published studies [24]. A phylogenetic tree was constructed using the neighbor-joining (NJ) method in MEGA11 (v11.0.13) with the Kimura 2-parameter model and pairwise deletion of gaps. Nodal support was evaluated with 1000 bootstrap replicates. Genotypes were assigned to each sample based on phylogenetic clustering with bootstrap support ≥ 70%. The thresholds of mean depth ≥ 10× and coverage ≥ 70% were chosen based on three considerations. First, the standardized RSV genotyping framework proposed by Goya et al. [25] recommends the G gene ectodomain as the minimum region for reliable phylogenetic clustering, providing a methodological basis for genotyping from partial gene regions. Second, a mean depth of 10× is widely adopted as a minimum standard for reliable variant detection and phylogenetic analysis in RSV whole-genome sequencing studies, including tiling amplicon-based assays that use >20× for high-confidence classification [26]. Third, in our own data, samples with mean depth below 10× or coverage below 70% showed markedly reduced assembly quality of the G gene HVR2 region, which was insufficient for reliable phylogenetic analysis. We therefore set ≥10× and ≥70% as the minimum quality control criteria.

### 2.5. Viral Metagenomic Identification and Codetection Analysis

Viral metagenomic analysis was performed using MapV (Metagenomics Analysis Pipeline for Virome, V1.0), an in-house pipeline developed in our laboratory [27,28]. In brief, the clean reads obtained in Section 2.3 were aligned to the human reference genome (GRCh38) using Bowtie2 (v2.3.5.1) to remove host-derived sequences. The remaining unmapped reads were assembled de novo using MEGAHIT (v1.2.9). No RSV-specific read removal was performed prior to de novo assembly; only host-derived reads were removed at this step. In parallel, the host-depleted clean reads were directly aligned to the same viral nucleotide and protein reference databases using BLASTn (v2.14.0) and DIAMOND (v2.1.8), respectively, under identical thresholds (E-value ≤ 1 × 10^−5^, identity ≥ 70%, query coverage ≥ 50%). Taxonomic assignment of read-level hits was performed with MEGAN6 (v6.24.23) using the same LCA algorithm. Thus, viral detection proceeded along two independent paths—direct read-level search and contig-level search—and the results of both were integrated by MVSM as described below. To eliminate residual non-viral microbial sequences, the assembled contigs were aligned against the NCBI non-redundant protein database (nr) using DIAMOND; contigs with best hits to cellular organisms (bacteria, fungi, protozoa, plants, and animals) were discarded, and the remaining contigs were retained for viral identification. These contigs were then searched against viral nucleotide and protein databases using BLASTn and DIAMOND, respectively, with an E-value cut-off of 1 × 10^−5^, minimum sequence identity of ≥70%, and query coverage of ≥50%. Taxonomic assignment was performed using MEGAN6 Metagenome Analyzer based on the lowest common ancestor (LCA) algorithm applied to the best BLAST hits. A multidimensional voting scoring model (MVSM) developed in-house was applied to assign a confidence score to each viral hit. Briefly, hits from both the read-level and contig-level searches that passed the annotation thresholds described above (E-value ≤ 1 × 10^−5^, sequence identity ≥ 70%, and query coverage ≥ 50%) were further evaluated against four independent criteria: (i) consistent recovery across multiple reference databases, (ii) abundance exceeding a sequencing-depth-normalized threshold, (iii) support from assembled contigs, and (iv) cross-sample consistency. Only taxa satisfying all four criteria were retained as positive detections. The scoring procedure and its performance are described in detail in our previous study [27].

To validate the metagenomic findings, we performed two levels of confirmation. First, in silico validation was conducted by re-searching the assembled viral contigs against the NCBI nucleotide database (BLASTn) to confirm taxonomic assignments. Second, selected viral detections—particularly those involving clinically relevant viruses or low-abundance hits—were experimentally confirmed by virus-specific PCR followed by Sanger sequencing of the amplicons from the original nasopharyngeal swab specimens. The pathogenic potential of the identified viruses was assessed by cross-referencing against the ViralZone database (https://viralzone.expasy.org, accessed on 13 July 2026) and the International Committee on Taxonomy of Viruses (ICTV) database; viruses listed as known human pathogens or as members of genera previously associated with human disease were classified as pathogenic. Codetection was defined as the detection of two or more distinct human viral taxa in the same specimen, each with a confidence score above the MVSM threshold and confirmed by in silico and experimental validation where feasible.

### 2.6. Identification and Phylogenetic Analysis of Codetected Viral Genomes

To further characterize the viral genomes identified in co-detected samples, we performed genomic annotation and phylogenetic analysis for each non-RSV virus detected. Open reading frames (ORFs) in the assembled viral contigs were predicted using the built-in ORF Finder in Geneious Prime (v2024.0.2) and validated by conserved domain searches against the NCBI Conserved Domain Database (CDD). For each contig, the best-matching viral reference sequences were identified by BLASTn (against the NCBI nucleotide database) and BLASTx (against the NCBI protein database) searches, based on nucleotide and amino acid sequence identity, respectively. Reference genomes from the same viral genus or family were selected according to the ICTV taxonomy, and multiple sequence alignments were performed using MAFFT (v7.526). Phylogenetic trees were reconstructed using the neighbor-joining (NJ) method with the Kimura 2-parameter model and 1000 bootstrap replicates in MEGA11 (v11.0.13). A viral contig was considered confidently identified when the following criteria were met: (i) predicted ORFs exhibited typical viral gene structures with conserved domains; (ii) BLASTn or BLASTx searches showed ≥90% nucleotide or amino acid identity to a known viral reference sequence; (iii) the contig phylogenetically clustered with the corresponding reference genome with bootstrap support ≥ 70%; and (iv) the taxonomic assignment was consistent with ICTV criteria for species and genus demarcation.

### 2.7. Statistical Methods and Visualization Tools

Statistical analyses and graphical presentations were performed using GraphPad Prism (v9.0; GraphPad Software, Boston, MA, USA). Continuous variables were summarized as mean ± standard deviation (SD) or median with interquartile range (IQR), as appropriate. Between-group comparisons were conducted using Student’s *t*-test or the Mann–Whitney U test for two groups, and one-way ANOVA or the Kruskal–Wallis test for multiple groups, depending on data distribution. Correlations between variables were assessed using Pearson’s or Spearman’s rank correlation coefficient as appropriate.

For comparisons of categorical variables—including associations between viral detection and demographic or clinical characteristics—Fisher’s exact test was applied due to small sample sizes and expected cell frequencies below 5 in several contingency tables. For paired comparisons of viral abundance between different viral taxa within the same samples, the Wilcoxon signed-rank test was used, as the abundance data (RPM values) were not normally distributed (Shapiro–Wilk test, *p* < 0.05) and exhibited considerable skewness. All statistical tests were two-sided, and *p* < 0.05 was considered statistically significant.

Heatmaps of viral metagenomic profiles and codetection matrix bubble plots were generated using the Hiplot online platform (https://hiplot.com.cn, accessed on 13 July 2026). Phylogenetic trees were constructed using MEGA11 (v11.0.13) and annotated and visualized using iTOL (https://itol.embl.de/, accessed on 13 July 2026).

## 3. Results

### 3.1. Participant Characteristics and Sequencing Output

This retrospective analysis included 42 children with laboratory-confirmed RSV infection (Table 1). The age distribution was as follows: 25 infants under 12 months, 5 toddlers between 1 and 2 years, and 12 children aged 2 to 12 years. Males accounted for 20 of the 42 cases and females for the remaining 22. Clinically, pneumonia was the most frequent diagnosis (17/42, 40.5%), followed by bronchopneumonia (10/42, 23.8%) and bronchitis (7/42, 16.7%); the remaining eight cases (19.0%) had incomplete clinical records or were identified through routine screening in the absence of overt respiratory symptoms. On qPCR re-testing, two specimens returned Ct values below 20, 11 gave values above 30, and the other 29 fell between 20 and 30.

Sequencing of the 42 libraries yielded 491.2 million raw reads in total (147.4 Gb), translating to a per-sample average of 11.7 million reads (95% CI: 10.5–12.9 million) or 3.5 Gb (95% CI: 3.1–3.9 Gb). To exclude the possibility of reagent or environmental contamination, a negative control (nuclease-free water) was processed in parallel with the clinical specimens through nucleic acid extraction, DNase treatment, reverse transcription, and MDA amplification. As it contained no amplifiable nucleic acid template, no library was obtained after library preparation and it was therefore not sequenced. This indicates that no exogenous amplifiable template was introduced during extraction and amplification, although it does not provide read-level information for contaminant filtering (see Section 4). In addition, the original laboratory records showed that only two of the 42 cases had an additional virus detected on initial clinical testing: one with *Human bocavirus* and one with enterovirus/rhinovirus. The other 40 cases were RSV mono-positive. Both viruses were also detected by metagenomic sequencing. Metagenomic sequencing further identified multiple viruses not covered by the routine panels; these were largely low-abundance viruses, including commensal or non-respiratory taxa, highlighting the broader discovery capacity of unbiased sequencing for codetection analysis.

### 3.2. RSV Genome Recovery and Genotyping

**Figure 2:**
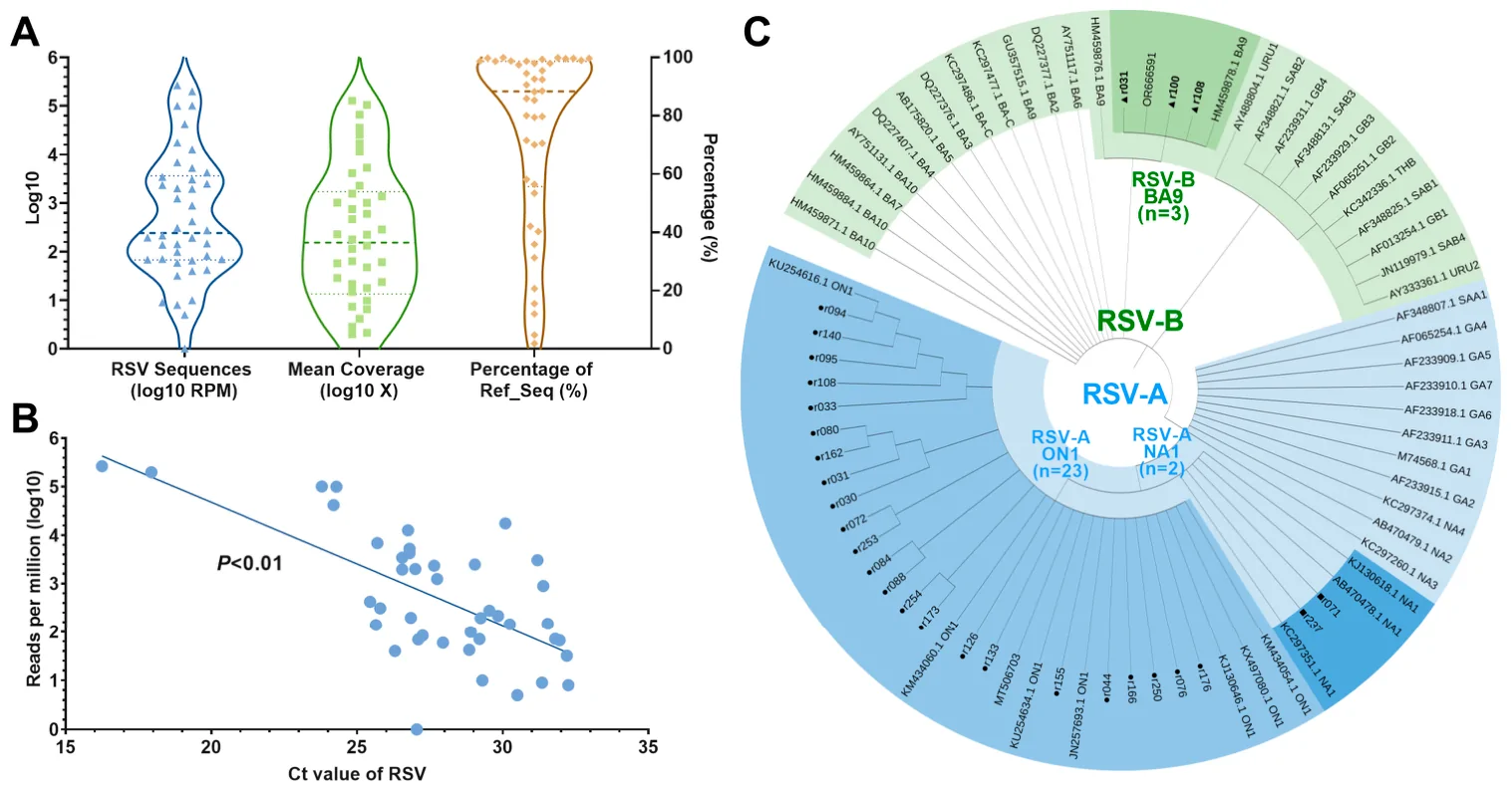
Metagenomic sequencing-based detection and genotyping of RSV. (**A**) Distribution of RSV-specific read counts (RPM), sequencing depth, and reference genome coverage across all samples. (**B**) Negative correlation between RSV-specific reads (RPM) and qPCR Ct values. (**C**) Phylogenetic tree of RSV based on G-gene HVR2 sequences. The tree was constructed using the neighbor-joining method in MEGA11. Colors and shapes of the symbols denote provisional genotypes based on clustering with reference sequences: RSV-A ON1-like (medium blue circles), RSV-A NA1-like (dark blue squares), and RSV-B BA9-like (green triangles). Genotype designations are preliminary and based on HVR2 analysis; full G gene or whole-genome sequencing would be required for definitive assignment under the updated classification framework [21].

All 42 samples yielded reads that aligned to the RSV reference genome (Figure 2A). Normalized to reads per million total reads (RPM), the median RSV-specific read count stood at 643.1 (95% CI: 290.1–4891). Median sequencing depth across the cohort was 155.9× (95% CI: 28.71–757.4), and the median fraction of the reference genome covered was 88.4% (95% CI: 71.5% to 97.5%). The number of reads mapping to RSV correlated inversely with qPCR Ct values (Figure 2B), consistent with the notion that pathogen-derived read counts from metagenomic data broadly reflect organismal abundance in the original specimen.

High-quality G-gene sequences were recovered from 26 of the 42 samples, yielding a total of 28 sequences—a discrepancy explained by two samples in which both RSV-A and RSV-B sequences were simultaneously present. Of these 28 sequences, phylogenetic analysis based on the HVR2 region showed that 25 clustered with RSV-A reference strains (23 clustering with ON1 and 2 with NA1 reference sequences) and 3 clustered with RSV-B reference strains (all clustering with BA9 reference sequences) (Figure 2C). These provisional genotype designations, assigned using the established HVR2-based approach, are consistent with the predominant RSV genotypes circulating in China in recent years. However, as noted in the Methods, definitive genotype assignment according to the current gold standard classification [21] would require full G gene or whole-genome sequences. Therefore, our HVR2-based results should be considered preliminary and interpreted with appropriate caution. Notably, the two samples with mixed subtype signals were confirmed by Sanger sequencing to carry both RSV-A ON1 and RSV-B BA9.

### 3.3. Metagenomic Profiling of Codetected Viruses and Codetection Patterns

**Figure 3:**
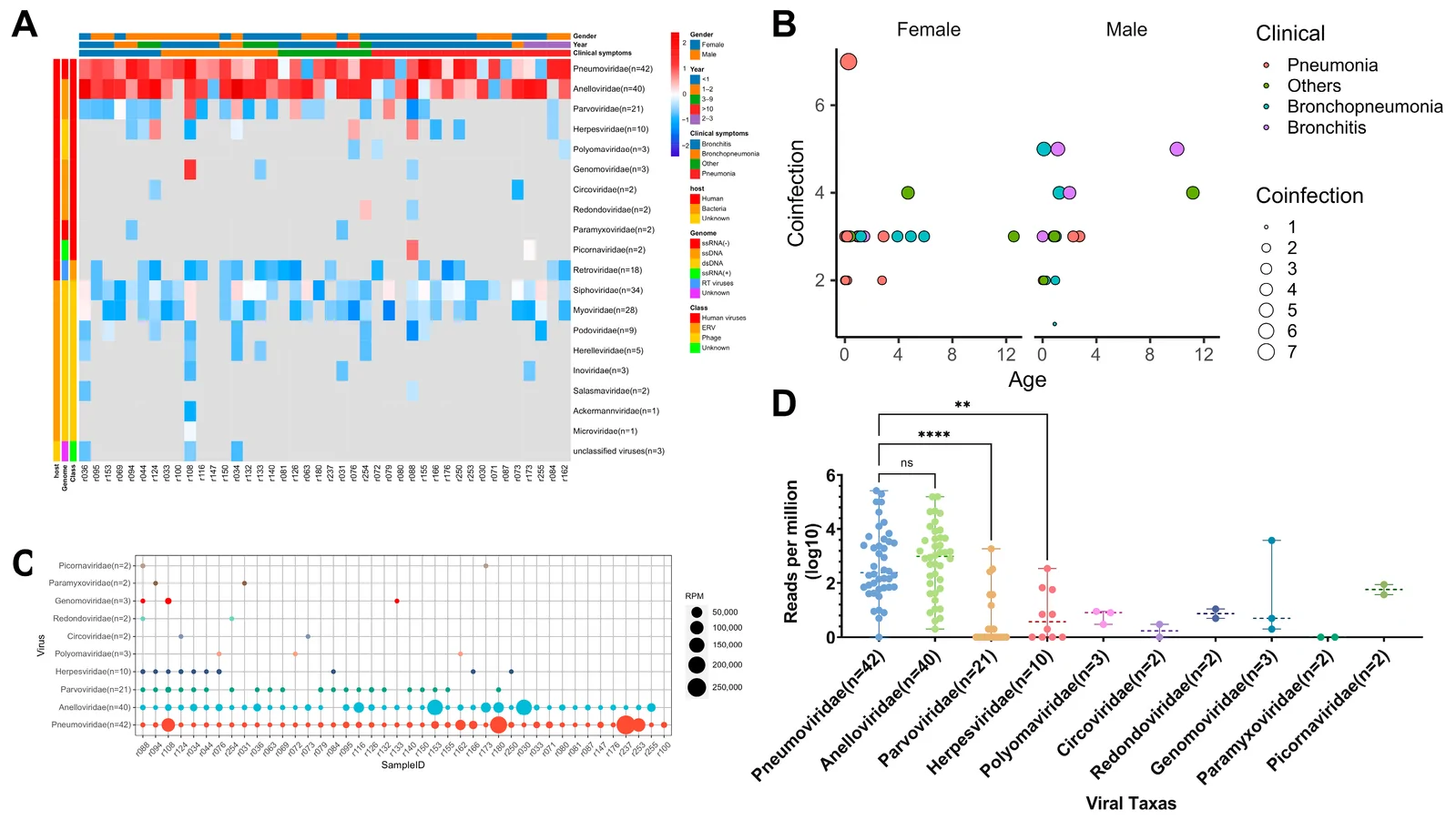
Metagenomic profiling of viruses co-occurring with RSV. (**A**) Heatmap of viral families detected in each sample. Columns represent individual cases; rows represent viral families. The left annotation bar indicates viral family classification, and the top annotation bar denotes patient characteristics (sex, age group, and clinical diagnosis). Color intensity reflects Z-score-normalized relative abundance (red: high; blue: low); gray indicates not detected. (**B**) Prevalence and relative abundance of human pathogenic viral families across the cohort. Circle size is proportional to the relative abundance (RPM) of each viral family. (**C**) Number of viral families detected per individual, stratified by sex, age group, and clinical presentation. (**D**) Comparison of relative abundances among the major viral families detected. “ns” indicates not significant (*p* > 0.05), “**” indicates *p* < 0.05, and “****” indicates *p* < 0.01.

Metagenomic analysis uncovered 20 viral families across the 42 specimens, 10 of which are known human pathogens. These included *Pneumoviridae* (present in all 42 samples), *Parvoviridae* (*n* = 21), *Paramyxoviridae* (*n* = 2), and *Herpesviridae* (*n* = 10) (Figure 3A,B). Detection of these viral families did not differ significantly by sex, age, or clinical diagnosis (Fisher’s exact test, all *p* > 0.05; Figure 3B).

In addition to the human viral pathogens described above, the heatmap in Figure 3A also shows sequences assigned to several bacteriophage families, including *Siphoviridae*, *Myoviridae*, and *Podoviridae*. Bacteriophages are common in the respiratory bacterial microbiota and have not been directly linked to human respiratory disease [29,30]. Their presence in our samples most likely reflects the bacterial communities of the respiratory tract rather than a pathogenic role. We therefore present them as background information and did not analyze them further. Further work specifically targeting respiratory bacteriophages may clarify their relationship with co-occurring respiratory pathogens [31].

The number of viral families per sample ranged widely: 30 specimens harbored at least three families (counting *Pneumoviridae*), 11 contained two, and just one yielded no detectable virus besides RSV (Figure 3C). The families most frequently found alongside RSV were *Anelloviridae* (*n* = 40) and *Parvoviridae* (*n* = 21); *Anelloviruses* are common in healthy children, and their clinical relevance remains unsettled. At the upper end, a single sample contained seven different viral families—among them *Pneumoviridae*, *Anelloviridae*, and *Parvoviridae*. Abundances varied markedly across taxa, with *Parvoviridae* and *Herpesviridae* present at levels well below those of *Pneumoviridae* (Wilcoxon signed-rank test, *p* < 0.05; Figure 3D).

**Figure 4:**
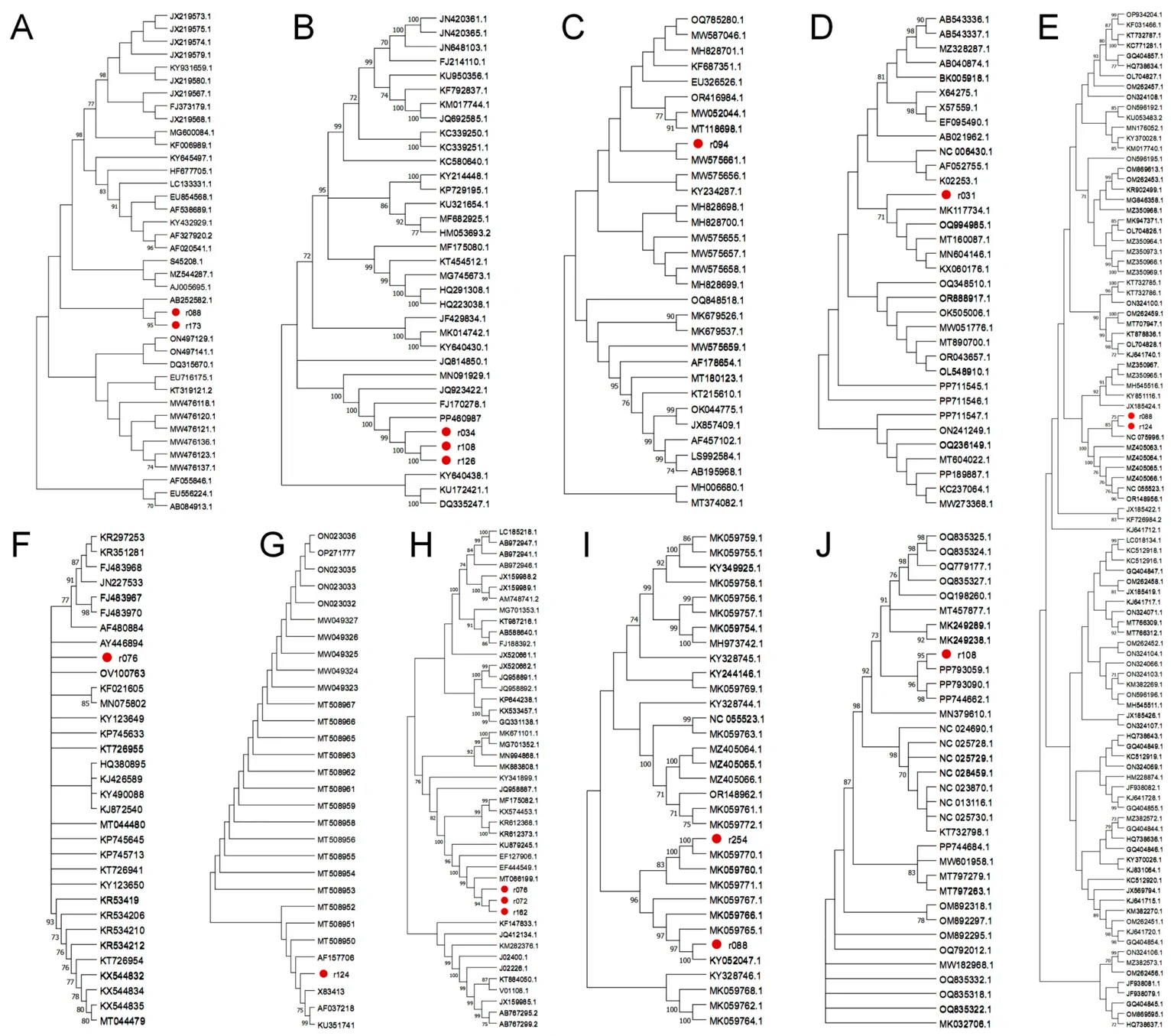
Phylogenetic relationships of co-infecting viruses based on whole-genome sequences (w) or key-gene nucleotide sequences (k). (**A**) *Picornaviridae* (k). (**B**) *Parvoviridae* (w). (**C**) *Paramyxoviridae*, genus Respirovirus (k). (**D**) *Paramyxoviridae*, genus *Orthorubulavirus* (k). (**E**) *Circoviridae* (w & k). (**F**) *Herpesviridae* (Cytomegalovirus/HHV-5, w). (**G**) *Herpesviridae* (Roseolovirus/HHV-6, k). (**H**) *Polyomaviridae* (w & k). (**I**) *Redondoviridae* (w & k). (**J**) *Genomoviridae* (k). Dots indicate sequences obtained from this study.

BLASTn/x searches against reference databases returned ≥ 90% nucleotide or amino acid identity to known viruses for most of the assembled sequences (Table 2). On the phylogenies, these sequences clustered consistently with established human viruses; we saw no signs of highly divergent or recombinant strains (Figure 4).

Across the 42 pediatric cases, we identified nine virus species or genera co-occurring with RSV. These fell into three broad groups according to transmission route and pathogenic profile. The first consisted of viruses with clear respiratory tropism and documented pathogenicity: *Human bocavirus* (HBoV, *n* = 21), *Human parainfluenza viruses* (HPIV, *n* = 2), and *Human parechovirus* (HPeV, *n* = 2). The second comprised viruses for which the pathogenesis is still poorly defined but that have been tentatively linked to respiratory illness—*WU polyomavirus* (WUPyV, *n* = 3), *Human oral-associated vientovirus* (*n* = 2), *PoSCV5-like circular viruses* (*n* = 2), and *Genomoviridae* (*n* = 3). The third group contained viruses that do not spread primarily through the respiratory route and are generally found as latent or commensal residents: *Human herpesviruses* (HHV, *n* = 10) and *Anelloviridae* (*n* = 41).

## 4. Discussion

In this study, we applied viral metagenomic sequencing to nasopharyngeal swabs from 42 children with RSV infection, providing an unbiased view of the viral codetection landscape in pediatric respiratory disease. Unlike targeted diagnostic approaches, metagenomic sequencing not only detected and genotyped RSV in every sample, but also revealed that mixed infections were the rule rather than the exception: 30 of 42 samples harbored at least three viral families, with up to seven families detected in a single specimen, and only one sample yielded no virus other than RSV. These findings challenge the single-pathogen diagnostic paradigm and suggest that the microbiological complexity of childhood respiratory infections has been substantially underestimated.

Viral codetections in respiratory specimens are not a new observation. The phenomenon has been recognized for more than three decades, first through culture-based studies and more recently through molecular diagnostics. With multiplex PCR panels now routine, codetection rates in pediatric populations have been extensively characterized and are known to vary considerably with age, clinical setting, enrollment criteria, and detection methods. Metagenomic studies have further revealed a rich viral diversity in respiratory samples, typically showing that known human pathogens predominate even in cases of unexplained illness. Previous metagenomic investigations of pediatric respiratory infections have reported similar patterns: known viral pathogens predominate even in cases of unexplained etiology [32], and the respiratory virome in Chinese children with severe acute respiratory infection is characterized by a limited set of viral families with *Anelloviridae* dominance in asymptomatic controls [33]. Our study differs from these earlier efforts in three respects. We focused specifically on RSV-confirmed cases, providing a detailed profile of the viral landscape surrounding the leading respiratory pathogen in young children. We also integrated RSV genotyping with codetection analysis, linking genetic variation in the primary virus to the polymicrobial context in which it occurs. And we identified several viruses—HBoV2 in respiratory specimens, HPIV-5, and less-characterized agents such as *Redondoviridae* and *Genomoviridae*—that are rarely picked up by routine testing, raising questions about their tissue tropism and possible clinical role. Thus, rather than simply reaffirming the high frequency of codetections, our study offers a comprehensive, untargeted view of the viral communities that accompany RSV, bringing into focus both the familiar and the frequently overlooked components of the pediatric respiratory virome.

The public health relevance of distinguishing between RSV-driven disease and bystander codetections has been underscored by recent real-world evidence from RSV immunoprophylaxis programs. The introduction of long-acting monoclonal antibodies (nirsevimab) into national immunization schedules in countries such as Spain and Chile has achieved high coverage and led to a dramatic reduction in RSV-associated hospitalizations among infants [34,35]. Notably, this single-pathogen intervention—which targets RSV alone—has been effective in preventing severe disease despite the frequent co-occurrence of other respiratory viruses documented in our study and elsewhere. If codetected viruses were major contributors to severe illness, one would expect that removing RSV alone would have a limited impact on overall disease burden. The observed reduction in severe lower respiratory tract infections, without evidence of a “replacement” effect whereby non-RSV viruses cause more severe illness, further supports the interpretation that RSV is the dominant driver of severe disease in most cases, with other detected viruses playing at most a contributory or bystander role [34,35]. These population-level findings complement our metagenomic observations and reinforce the clinical rationale for prioritizing RSV-specific prevention strategies, even in settings where viral codetections are common.

The clinical value of detecting multiple viruses in a given patient should not be taken for granted. An alternative view, grounded in resource-conscious practice, holds that expanding diagnostic testing beyond what is strictly needed may add cost and complexity without clear benefit to patients. Meskill and colleagues, for instance, analyzed more than 13,000 respiratory specimens and found that influenza virus was rarely detectable in children who already tested positive for RSV, leading them to question the routine use of influenza testing in RSV-positive children [36]. This observation underscores a key tension: metagenomic sequencing offers an unrivaled view of the viral landscape, yet its routine use in clinical practice must be balanced against cost, turnaround time, and the difficulty of interpreting incidental findings. Many codetected viruses may be bystanders or commensals, and their detection could prompt unnecessary concern, additional workup, or even inappropriate treatment. We therefore do not advocate universal metagenomic testing. Instead, we view our findings as hypothesis-generating—they reveal the polymicrobial complexity of RSV infections and highlight the need for prospective studies that link codetection patterns to clinical outcomes, so that we can learn which codetections matter and which do not.

However, interpreting the clinical significance of these codetections is not straightforward. Several scenarios can explain why two or more viruses are found in the same specimen. The first is true co-infection, meaning that two or more viruses are actively replicating and each contributes to the disease. The second is sequential infection or superinfection, where one virus was acquired shortly after another, but only the later or more dominant virus accounts for the current illness. The third is a primary infection accompanied by incidental carriage or colonization of additional viruses that do not affect symptoms. A single cross-sectional nucleic acid test cannot readily distinguish among these possibilities, yet the distinction matters for both clinical decision-making and epidemiological interpretation.

Among the established respiratory pathogens, *Human bocavirus* (HBoV) was the most frequently detected virus co-occurring with RSV (21/42, 50%). HBoV1 is widely recognized as a respiratory pathogen, yet its independent pathogenicity remains debated, largely because of its high co-detection rate with other respiratory viruses [37]. This debate is further fueled by studies showing that HBoV and other viruses, including human coronaviruses, are detected in asymptomatic children as often as, or even more frequently than, in symptomatic patients with pneumonia [12,13]. Such findings argue that the mere presence of viral nucleic acids—particularly in the upper respiratory tract—may represent colonization, asymptomatic carriage, or residual shedding from a prior infection, rather than active disease causation. Therefore, when a dominant pathogen such as RSV is concurrently detected, HBoV positivity may often reflect coincidental carriage with limited clinical significance. This interpretation is consistent with systematic review evidence indicating that the presence of two or more viruses is generally not associated with worse clinical outcomes than single infections [14], and it cautions against assuming additive or synergistic pathogenic effects among codetected viruses. Collectively, these observations support the view that, in the majority of cases in this cohort, the clinical illness was likely driven primarily by RSV, with other detected viruses playing at most a bystander role. Notably, we recovered near-complete genomes of HBoV2 from three respiratory samples, which shared 99.6% nucleotide identity with the BJQ435 strain (JX257046) originally isolated from a child with acute diarrhea. HBoV2–4 have traditionally been considered enteric viruses [38]; their presence in respiratory specimens in our study suggests that the tissue tropism of these genotypes may be broader than previously appreciated. We also identified two HPIV infections (HPIV-3 and HPIV-5) and two HPeV infections. Of these, HPIV-5 remains poorly characterized in relation to human disease [39], and its clinical relevance deserves further investigation. In one sample, a single read mapped to *rhinovirus* (RV) fell below our detection threshold and was excluded—an observation that underscores the sensitivity limits of metagenomic sequencing: low-abundance pathogens may require deeper sequencing or targeted enrichment strategies for reliable detection.

We also detected several less-characterized viruses. *WU polyomavirus* (WUPyV) was present in three samples; previous studies have reported its detection in children with acute respiratory infections in China, with prevalence estimates varying across different regions and study populations [40,41], but its pathogenesis remains obscure. Human oral-associated vientovirus (two samples), a member of the *Redondoviridae*, has been linked to periodontal disease [42,43] and is frequently recovered from respiratory specimens, yet its tissue tropism and pathogenic potential are still uncertain. *Genomoviridae* (three samples) are a family of viruses identified by metagenomic sequencing from diverse environmental, plant, animal and human sources; one genus, *Gemykibivirus*, has been tentatively associated with severe respiratory infections [44]. In addition, PoSCV5-like circular viruses (two samples), belonging to the *Circoviridae*, were originally described in respiratory secretions from a patient with unexplained febrile illness [45], but their role in respiratory pathology remains unclear. These four virus groups are either zoonotic or environmentally acquired, and their clinical significance as respiratory coinfecting agents warrants close monitoring.

Among viruses not transmitted primarily via the respiratory route, we identified human herpesviruses in 10 samples—five HHV-5 (*cytomegalovirus*) and five HHV-6. Both are *Betaherpesviruses* that typically establish latent infections and reactivate during immunosuppression. Although not classic respiratory pathogens, they can be shed via saliva or respiratory droplets, and their potential synergistic effects with RSV deserve further attention. *Anelloviridae* were detected in 41 samples (97.6%), making them the most frequent viruses co-occurring with RSV. However, *Anelloviruses* are highly prevalent in healthy children and are generally regarded as commensals rather than overt pathogens; their high detection rate likely reflects the normal composition of the pediatric respiratory virome rather than a specific pathogenic signal. Distinguishing between high prevalence and pathogenic contribution is therefore critical when interpreting the clinical relevance of codetections.

This study has several limitations that should be considered when interpreting the findings. The sequencing depth, approximately 3.5 Gb per sample, fell within the range commonly used in virome surveys, yet it may have been insufficient to reliably detect very low-abundance pathogens. In addition, the MDA step used for non-targeted cDNA amplification may introduce sequence-dependent bias, which could affect the semi-quantitative interpretation of viral abundance. Although we validated selected detections by PCR and Sanger sequencing, future studies employing quantitative standards or spike-in controls would help to more rigorously assess the quantitative performance of MDA-based metagenomic workflows. The single rhinovirus read that fell below our detection threshold serves as a cautionary example. A further limitation is that our negative control did not proceed to sequencing, and therefore could not be used to identify and subtract contaminant taxa at the bioinformatic level. Future studies should include full-process controls carried through library preparation and sequencing, following contamination-control frameworks such as that proposed by Ibañez-Lligoña et al. [46]. Deeper sequencing or targeted enrichment strategies could improve sensitivity for such low-titer pathogens. Our cross-sectional design likewise prevents us from distinguishing between true co-infection, superinfection, and chronic viral carriage. This distinction is particularly challenging when detection relies solely on nucleic acid amplification or sequencing, as viral RNA or DNA may persist for weeks after clinical resolution, as has been well documented for SARS-CoV-2 and other respiratory viruses. Because the upper respiratory tract is continuously exposed to environmental microbes, we cannot rule out the possibility that some of the detected viral sequences, particularly those from typically enteric or environmental viruses, reflect transient colonization, asymptomatic carriage, or residual nucleic acids from resolved infections rather than active lower respiratory infection. A related consideration is that our analysis focused exclusively on viruses. Although bacterial reads were incidentally captured during host-depletion and viral identification, they were not systematically classified or quantified in this study. The interplay between viruses, bacteria, and host immunity is known to modulate clinical outcomes [47], and future studies that integrate viral and bacterial metagenomic profiling with host transcriptomics will be essential to disentangle these cross-kingdom interactions. Indeed, previous studies have shown that certain viral combinations, such as *rhinovirus* and *influenza A virus*, may attenuate disease severity through viral interference [48,49], suggesting that the clinical impact of multiple viral codetections is not simply additive. Throughout this manuscript, we therefore use the term “codetection” to describe the presence of multiple viral nucleic acids in a specimen, acknowledging that this finding does not necessarily equate to concurrent active infection. Future prospective studies incorporating viral culture, serology, quantitative viral load dynamics, or host transcriptional signatures will be required to resolve this ambiguity and to determine which codetections are clinically meaningful.

It is also worth being explicit about the limits of what these data can tell us. Our workflow documents viral nucleic acids in a specimen, and it identifies which viruses are present and roughly how abundant they are, but it says nothing about whether those viruses are replicating, transcriptionally active, or infectious. A signal may therefore come from an actively replicating virus, from nucleic acid left over after a resolved infection, from asymptomatic carriage, or from non-viable virions. The negative correlation between RSV read counts and qPCR Ct values indicates that read abundance tracks template abundance, but that is not the same as replication. Nor can our design resolve the scenarios outlined above. Resolving these questions would require viral culture, serology, serial viral load measurements, or host transcriptional profiling, none of which our study design includes. For this reason we report all findings as co-detections and make no claim about active coinfection or clinical causality.

Our RSV genotyping warrants a methodological caveat. We used the G gene HVR2 region for phylogenetic analysis—a marker widely adopted in RSV molecular epidemiology, but one that does not offer the same resolution as longer G gene fragments or complete genomes. We recognize that the standardized classification framework recently published by Goya et al. [21]. represents the current gold standard for RSV genotype assignment below the subgroup level, which recommends at least the full ectodomain or the entire G gene for robust phylogenetic classification. However, due to the metagenomic design of our study, the HVR2 region was the most consistently recoverable region across samples. With that in mind, our HVR2-based analysis can only provide a tentative view of the genotype distribution. The observation that ON1 and BA9 predominated in our cohort is consistent with what has been reported in China over the past decade, but we emphasize that these assignments are preliminary. Confirmation under the new classification framework, as well as more refined tracking of strain evolution, will depend on future work using full G gene or whole-genome sequences.

Despite these limitations, our study provides a comprehensive, untargeted view of the viral communities accompanying RSV infection in children. The results confirm that while codetections are frequent, the additional viruses detected—with the exception of HBoV, HPIV, and HPeV in a limited number of cases—were present at substantially lower abundance than RSV. The additional viruses that could be considered clinically relevant (HBoV, HPIV, and HPeV) fall within the coverage of conventional multiplex PCR panels, whereas the viruses uniquely detected by metagenomics were largely low-abundance, commensal, or non-respiratory taxa of uncertain clinical significance. This raises important questions about the added clinical value of metagenomic sequencing in this context. Rather than advocating for its routine use, we view our findings as hypothesis-generating: they illuminate the polymicrobial complexity of RSV infections and highlight the need for prospective studies that link codetection patterns to clinical outcomes. Only when such studies establish which codetections are truly clinically meaningful can the potential role of metagenomic sequencing in routine diagnostics be properly evaluated.

## Figures and Tables

**Table 1:** Basic Characteristics of Cases and Real-Time PCR Detection Results for RSV in This Study.

		Gender		Sum
		Male	Female	
Age (years)	<1	13	12	25
	1–2	2	3	5
	2–3	2	2	4
	3–9	2	4	6
	>10	1	1	2
Collection Time	November 2025 to December 2025	7	5	12
	January 2026 to March 2026	10	4	14
	April 2026 to May 2026	3	13	16
Clinical Symptoms	Pneumonia	5	12	17
	Bronchopneumonia	6	4	10
	Bronchitis	5	2	7
	Others	4	4	8
RSV-Ct	<20	2		2
	20–30	16	13	29
	>30	2	9	11
Total		20	22	42

**Table 2:** Identification Results of Viral Genomes Codetected with RSV in This Study.

Viral Family	Genome Type (RNA/DNA)	Viral Genus	Viral Species	No. Cases	High Quality Genome *	% of Ref Seq	Mean Coverage	Nucleotide Ref	Nucleotide Identity	Amino Acids Ref	Amino Acids Identity
*Picornaviridae*	RNA	*Parechovirus*	*Human parechovirus 1*	2	2	6.0–6.1	9.3–33.0	MG026489	95.1	- **	-
*Parvoviridae*	DNA (ssDNA)	*Bocaparvovirus*	*Human bocavirus 2*	21	3	98.6–100	27.9–1095.5	JX257046	99.6	AFW98868	99.1
*Paramyxoviridae*	RNA	*Orthorubulavirus*	*Parainfluenza virus 5*	1	1	1.7	0.1	OQ994985	100	WLQ70500	100
		*Respirovirus*	*Human respirovirus 3*	1	1	30	0.6	MW575661	98.3	WZX10052	98.5
*Herpesviridae*		*Cytomegalovirus*	*Human betaherpesvirus 5*	5	1	95.9	16.4	KP745697	100	APG57520	99.8
	DNA (dsDNA)	*Roseolovirus*	*Human betaherpesvirus 6*	5	1	12.3	0.3	OP271777	99.9	AVK93191	99.5
*Polyomaviridae*	DNA (dsDNA)	*Betapolyomavirus*	*WU Polyomavirus*	3	3	70.0–97.9	1.4–6.8	KX650191	98.2–99.9	ADD51108	87.5
*Redondoviridae*	DNA (ssDNA)	*Torbevirus*	*Human oral-associated vientovirus*	2	2	34.4–93.9	0.7–23.9	MK059770	92.5–99.1	QCD25332	78–94.2
*Genomoviridae*	DNA (ssDNA)	*-*	*Genomoviridae* sp.	3	1	88.9	4898.9	PP793059	96.2	XCO47943	97.6
*Circoviridae*	DNA (ssDNA)	*-*	*Human PoSCV5-like circular virus*	2	2	60.5–99.2	12.6–48.6	NC_075996	91.8–95.7	YP_010796351	89.2–92.7

* Virus genomes or major structural genes with coverage > 70% were assigned to the closest reference sequences by BLAST searches. ** Sequences marked with ** fall within non-structural gene regions.

## Data Availability

The data presented in this study are available on request from the corresponding author, because the raw data contain personal information of pediatric patients.
